# Impact of facial angiofibromas in tuberous sclerosis complex and reported efficacy of available treatments

**DOI:** 10.3389/fmed.2022.967971

**Published:** 2022-08-29

**Authors:** Marie Monaghan, Pooja Takhar, Luke Langlands, Markus Knuf, Sam Amin

**Affiliations:** ^1^Department of Paediatric Neurology, University Hospitals Bristol NHS Foundation Trust, Bristol, United Kingdom; ^2^Tuberous Sclerosis Association, Epsom, United Kingdom; ^3^Children's Hospital and Tuberous Sclerosis Centre, Worms, Germany; ^4^Tuberous Sclerosis Germany (TSDEV), Wiesbaden, Germany

**Keywords:** tuberous sclerosis, facial angiofibromas, rapamycin, sirolimus, TSC

## Abstract

Tuberous Sclerosis Complex (TSC) is a genetic condition which leads to a loss of inhibition of cellular growth. Facial angiofibromas (FAs) are hamartomatous growths associated with TSC that appear as multiple small, erythematous papules on the skin of the face and may resemble more severe forms of acne vulgaris. FAs have been reported in up to 74.5% of pediatric TSC patients, rising to up to 88% in adults >30 years old. They have not been closely studied, potentially overshadowed by other, systemic features of TSC. To investigate the impact of FAs, a common clinical feature for patients with TSC, we performed a non-interventional study in the form of a survey, completed by people living with TSC and FAs, or their caregiver as a proxy, if necessary. Patients were recruited via patient organizations in the UK and Germany. Data was received from 108 families in the UK (44 patients, 64 caregivers) and 127 families in Germany (50 patients, 64 caregivers). Exclusion criteria were those outside of 6-89 years, those without FAs, or those enrolled in a clinical trial. Where caregivers reported on behalf of an individual unable to consent, they were required to be adults (>18 years). Patient experience in the design of the survey was considered from practical and logistical perspectives with survey questions assessing multiple aspects relating to FAs including age of onset, perceived severity, treatments, perceived efficacy of treatments and perceived psychosocial impacts of the FAs. The psychosocial impacts of FAs for the individuals as well as for caregivers were explored in terms of social, occupational and leisure activities. Results of the survey demonstrated that for those with TSC-related moderate or severe FAs, there is an impact on quality of life and psychosocial impacts in the form of anxiety and depression. This finding was also noted by caregivers of TSC individuals in these categories. The treatment most frequently received to improve FAs, topical rapamycin/sirolimus, was found to be successful in the majority of those who received it.

## Introduction

Tuberous Sclerosis Complex (TSC) is a rare, autosomal dominant genetic condition relating to mutations in either *TSC1* or *TSC2*, which code for hamartin and tuberin proteins, respectively, leading to constitutive activation of the mammalian Target of Rapamycin Complex 1 (mTORC1) and consequently a heterogenic loss of inhibition of cellular growth. This loss of inhibition of cellular growth leads to the development of benign tumors in the brain and other vital organs, such as the kidneys, heart, liver, eyes, lungs and skin (1). The central nervous system is typically involved, which may result in associated neuropsychiatric disorders such as cognitive impairment, autism, and other behavioral disorders—known as ‘TSC-Associated Neuropsychiatric Disorders' (TAND), in addition to other neurological symptoms, such as seizures (2).

Functionally, the proteins hamartin and tuberin form a complex which regulates cellular growth, therefore loss of function mutations lead to dysregulated growth. *TSC2* mutations account for the majority of TSC cases and are associated with more severe symptoms (3). Disease prevalence is estimated to be 7–12 in 100,000 (4).

While TSC is a highly variable condition with inter-patient variability in signs, symptoms, and severity, skin abnormalities are common. Facial angiofibromas (FAs) are thought to occur in up to 88% of TSC patients (5, 6). These lesions, composed of blood vessels and fibrous tissue, have previously been reported to typically appear after the age of 5 years, often preceded by facial flushing (5).They are considered one of the key diagnostic criteria for TSC (7).

The facial appearance resulting from FA lesions is associated with high psychological and physical morbidity (for example, recurrent bleeding or nasal obstruction) (8), as such it may be considered alongside acne vulgaris, port wine stains, atopic dermatitis, congenital melanocytic nevi and other conditions as a psychodermatological condition (9) with both physical and psychosocial impacts. The onset of facial dermatological conditions during school-age and adolescence has been found to cause a particularly negative psychsocial impact at a time when peer relationships gain importance and self-concept matures (10). Such is the impact of psychodermatological conditions upon patients' self-esteem that asking patients or their caregivers to rate the satisfaction with their skin on a scale from 1 to 10 has been suggested (11).

Traditionally, treatment options have focused on removal, such as surgical or laser procedures. According to the “Updated TSC International Diagnostic Criteria and Surveillance and Management Recommendations” (7), intervention with mTOR inhibitors (mTORis), pulsed dye or ablative lasers, or surgical excision can be appropriate for lesions that are large, disfiguring, prone to bleeding, or painful. Less invasive topical treatment with mTORis (rapamycin/sirolimus gels) has been advocated and its safety and efficacy for this purpose has been demonstrated in clinical trials (12, 13).

Jansen and colleagues (14) reported a substantial burden of TSC on the personal lives of individuals with TSC and their families. Nearly half of the patients experienced negative progress in their education or career due to TSC; additionally, many of their caregivers were unemployed resulting from the time commitment associated with the care they provided. Most caregivers indicated that TSC affected family life, and social and working relationships. Furthermore, well-coordinated care was considered difficult to access, and patients experienced moderate rates of pain or discomfort as well as anxiety or depression (14).

TSC patients may be challenged across multiple body systems as a result of having multiple organ hamartomas. Such effects can present challenges for the patients themselves as well as their caregivers. There are some studies which have explored the quality of life and the burden of TSC related illness reported by patients and their caregivers (15). However, there is very little reported relating to the severity and psychosocial impacts of TSC-associated FAs in particular, for the affected individuals and their caregivers which this study aimed to establish.

## Materials and methods

This was a non-interventional, observational study consisting of a cross-sectional online survey with 17 questions (see Table 1).

**Table 1:** Data subjects' responses to sample characteristics (Q1–Q8 of survey).

	**Base** **(*n* = 235)**	**UK** **(*n* = 108)**	**Germany** **(*n* = 127)**
**Q1 Which of the following specialists treat you for your FA?**			
Dermatologist	113 (48%)	55 (51%)	58 (46%)
TSC clinic	97 (41%)	49 (45%)	48 (38%)
Neurologist	68 (29%)	23 (21%)	45 (35%)
Primary care provider	50 (21%)	18 (17%)	32 (25%)
Other	30 (13%)	11 (10%)	19 (15%)
Pediatrician	29 (12%)	10 (9%)	19 (15%)
**Q2 On average, how often do you go to a TSC clinic?**			
Once a year	113 (48%)	62 (57%)	51 (40%)
Never	83 (35%)	35 (32%)	48 (38%)
Every quarter	35 (15%)	9 (8%)	26 (20%)
Monthly or more often	4 (2%)	2 (2%)	2 (2%)
**Q3 How severe would you say your FAs are?**			
Mild	71 (30%)	32 (30%)	39 (31%)
Moderate	120 (51%)	56 (52%)	64 (50%)
Severe	38 (16%)	16 (15%)	22 (17%)
Unsure	6 (3%)	4 (4%)	2 (2%)
**Q4 How old were you when FAs first appeared?**			
<1 year	17 (7%)	8 (7%)	9 (7%)
1–2 years	32 (14%)	17 (16%)	15 (12%)
3–5 years	88 (37%)	39 (36%)	49 (39%)
6–10 years	55 (23%)	22 (20%)	33 (26%)
11–15 years	26 (11%)	11 (10%)	15 (12%)
16–18 years	6 (3%)	5 (5%)	1 (1%)
18+	11 (5%)	6 (6%)	5 (4%)
**Q5 How significant are your FAs to your quality of life compared with your other symptoms related to TSC?**			
The most notably significant	32 (14%)	17 (16%)	15 (12%)
Notably significant	51 (22%)	16 (15%)	35 (28%)
Moderately notable or significant	64 (27%)	33 (31%)	31 (24%)
Mildly notable or significant	66 (28%)	33 (31%)	33 (26%)
Not at all notable or significant	22 (9%)	9 (8%)	13 (10%)
**Q6 What, if any, treatments or medicines have you received to improve your FAs?**			
Topical rapamycin/sirolimus	106 (45%)	51 (47%)	55 (43%)
Laser ablation	80 (34%)	35 (32%)	45 (35%)
No treatment	47 (20%)	20 (19%)	27 (21%)
Treatment of the underlying genetic syndrome	33 (14%)	6 (6%)	27 (21%)
Other	32 (14%)	17 (16%)	15 (12%)
Electrodissection	16 (7%)	4 (4%)	12 (9%)
I am not sure	3 (1%)	1 (1%)	2 (2%)
**Q7 How successful would you say your treatment(s) have been?**			
Very successful, they have made a noticeable improvement to the FAs	49 (26%)	17 (20%)	32 (33%)
Somewhat successful and have reduced the FAs	68 (37%)	40 (46%)	28 (29%)
Not very successful, a slight improvement of the FAs	55 (30%)	22 (25%)	33 (34%)
Very unsuccessful, no difference to the FAs	13 (7%)	8 (9%)	5 (5%)
**Q8 How well does your treatment/do your treatments help your FAs?**			
1 (The treatment does not help at all)	15 (8%)	9 (10%)	6 (6%)
2	25 (14%)	14 (16%)	11 (11%)
3	63 (34%)	23 (26%)	40 (41%)
4	44 (24%)	24 (28%)	20 (20%)
5 (The treatment helps considerably)	38 (21%)	17 (20%)	21 (21%)

People living with FAs were recruited through invitations distributed *via* Patient Organizations (POs) in monthly TSC newsletters, targeted mailing lists, and social media channels in the UK (Tuberous Sclerosis Association—www.Tuberous-Sclerosis.org) and Germany (Tuberöse Sklerose Deutschland e.V.—www.TSDEV.org). The invitations described a voluntary, unpaid, structured online survey of approximately 15 minutes in duration. Data subjects accessed the survey *via* an English language or a German language link distributed by the respective POs.

The survey materials were originally developed in English and then translated into German, and certified by an accredited translation agency. While external pilot testing of the survey with patients was not performed, internal quality checks ensured functionality and the survey was reviewed by members of UK and German POs.

The Survey included demographics and a series of questions evaluating the impact of FAs on quality of life (see Table 1). It was accessible for over 4 months (September to October 2021). There was no follow-up.

Inclusion criteria were:

- Person or caregiver of a person with a diagnosis of TSC.
- Aged 6–89 years.
- If a young adult: capacity to consent to a study (per national regulations for each country).
- If a caregiver was responding on behalf of a young subject (<18 years) the caregiver needed to be an adult (>18 years).

The exclusion criterion was being concurrently enrolled in another clinical trial.

All data subjects provided informed consent prior to completing the survey and were informed of their rights under General Data Protection Regulations (GDPR) as well as national, regional and local laws pertaining to privacy and data protection. Data subjects were informed that the survey was sponsored (Plusultra pharma).

The study was performed in compliance with the European Pharmaceutical Market Research Association (16) and was approved by the ethics committee of the Western Institutional Review Board (Tracking number: 20214481). Patients remained anonymised with de-identified patient information collated and aggregated. The survey consisted of eligibility screening questions followed by survey questions for eligible subjects. The screening questions verified that: there had been a confirmed diagnosis of TSC; the person completing the survey was either a patient or caregiver; the patient's age was within the stated criteria (and if 13–17 years old, considered suitable to participate); and that the caregiver's age was within the stated criteria.

## Results

**Figure 1:**
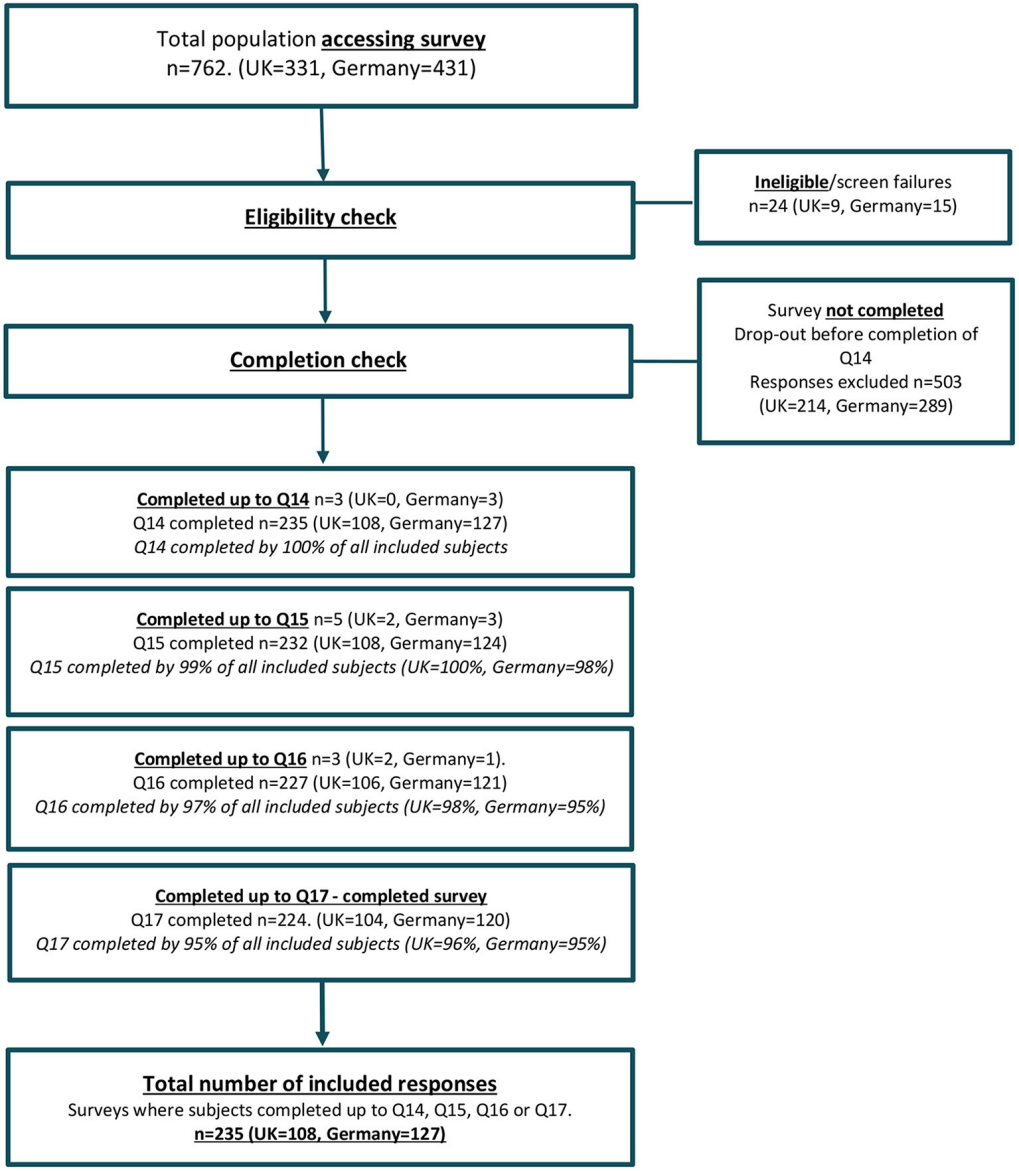
Population tree.

The survey was accessed by 762 (UK = 331, Germany = 431, see Figure 1). There were 24 screen failures (UK = 9, Germany = 15). Screen failures were predominantly due to the survey being attempted by a non-patient or non-caregiver (*n* = 12, UK = 6, Germany = 6). This reason was followed by a lack of confirmed TSC diagnosis (*n* = 7, UK = 2, Germany = 5). Finally, some caregivers (*n* = 4, UK = 1, Germany = 3) cited their age as <18 years while another respondent declined consent (Germany = 1).

After five screening questions, 503 data subjects did not complete the survey to at least question 14 (UK = 214, Germany = 289); approximately twice as many as those who did (*n* = 235). Most survey drop-outs (*n* = 489; 97%) did so in the first five questions after screening, which assessed sample characteristics (see Table 1). The reason for not completing the survey is not known.

The survey was completed to at least question 14 by 235 eligible subjects (UK = 108, Germany = 127). A total of 94 patients (UK = 44, Germany = 50) and 141 caregivers (UK = 64, Germany = 77) were included. The age of the patients with TSC ranged from 6 years (per eligibility criteria) up to 70 years. The mean age was 30 years (UK = 31, Germany = 29) with a standard deviation of 15 years (UK = 16, Germany = 14). Approximately a quarter (23%) were pediatric (55/235 aged 6–17 years) and thus this group contributed either *via* proxy if <13 years or directly if aged 13–17 and considered suitable to complete the survey independently.

The subjects deemed eligible for analysis included 11 data subjects (2 patients, 9 caregivers) who had not completed the full survey (up to and including Q17) but had completed a sufficient majority of questions (up to and including Q14). These 11 subjects were included for analysis of their completed questions, with the variable sample size being reported for each analysis (see Figure 1).

Descriptive analyses were conducted in IBM^®^ SPSS^®^ Data Collection Survey Reporter v7.5 software. The descriptive statistics varied according to the type of data being described. Categorical data was analyzed for base size, frequencies and percentages. Continuous data was reported as base size, mean and median averages, standard deviations, interquartile ranges, minimum and maximum values and 95% confidence intervals.

Results for patients with TSC living with FAs will be reported as “data subjects;” combining patient and caregiver/proxy responses. In reference to treatment, all responses are in relation to the affected individual with TSC aside for Q16–17 which were directed toward caregivers (see Tables 6, 7). Between the UK and Germany, similar proportions of data subjects were patients; 41% for the UK (44/108), 39% for Germany (50/127).

Questions relating to the study population's characteristics (see Table 1) identified that the three most common specialists or clinics attended were comparable between the UK and Germany. Combined, they were: Dermatologist (48%), a TSC clinic (41%) and neurologist (29%), with 48% visiting a TSC clinic at least annually. The reported severity of TSC-associated FAs was comparable between the two countries; 51% (CI: 41.7,60.2) reporting moderate FAs, 30% (CI: 19.7, 42.0) reporting mild FAs and 16% (CI: 6.2, 31.5) reporting severe FAs, with a small minority (~3%) undecided. The most common age for FAs to appear was 3–5 years old (37%), followed by 6–10 years old (23%). More common than other treatments, data subjects (45%) reported treatment for FAs with topical rapamycin/sirolimus (whether alone or in combination with other treatments). This was followed by laser ablation (34%). Of those treated with topical rapamycin/sirolimus, 67% (CI: 57.2, 75.8) found the treatment “very” or “somewhat successful” (see Table 2). This was higher than for those who had not received topical rapamycin/sirolimus where 59% reported successful/somewhat successful response to treatment.

**Table 2:** Treatment success for people who had received topical rapamycin/sirolimus with and without other common treatment options for Fas.

	**Topical rapamycin/sirolimus (with/without any other treatment)**	**Topical rapamycin/ sirolimus only**	**Topical rapamycin/sirolimus and at least one other treatment**	**Received at least one other treatment but not with Topical rapamycin/sirolimus**	**No treatment/ don't know**
	**(*n* = 106)**	**(*n* = 64)**	**(*n* = 42)**	**(*n* = 79)**	**(*n* = 50)**
**Q7 How successful would you say your treatment(s) have been?**					
Very successful, they have made a noticeable improvement to the FAs	26 (25%)	15 (23%)	11 (26%)	23 (29%)	–
Somewhat successful and have reduced the FAs	44 (42%)	27 (42%)	17 (40%)	24 (30%)	–
Not very successful, a slight improvement of the FAs	28 (26%)	15 (23%)	13 (31%)	27 (34%)	–
Very unsuccessful, no difference to the FAs	8 (8%)	7 (11%)	1 (2%)	5 (6%)	–
**Q8 How well does your treatment/do your treatments help your FAs?**					
1 (The treatment does not help at all)	8 (8%)	6 (9%)	2 (5%)	7 (9%)	–
2	16 (15%)	11 (17%)	5 (12%)	9 (11)	–
3	35 (33%)	19 (30%)	16 (38%)	28 (35%)	–
4	28 (26%)	14 (22%)	14 (33%)	16 (20%)	–
5 (The treatment helps considerably)	19 (18%)	14 (22%)	5 (12%)	19 (24%)	–

**Figure 2:**
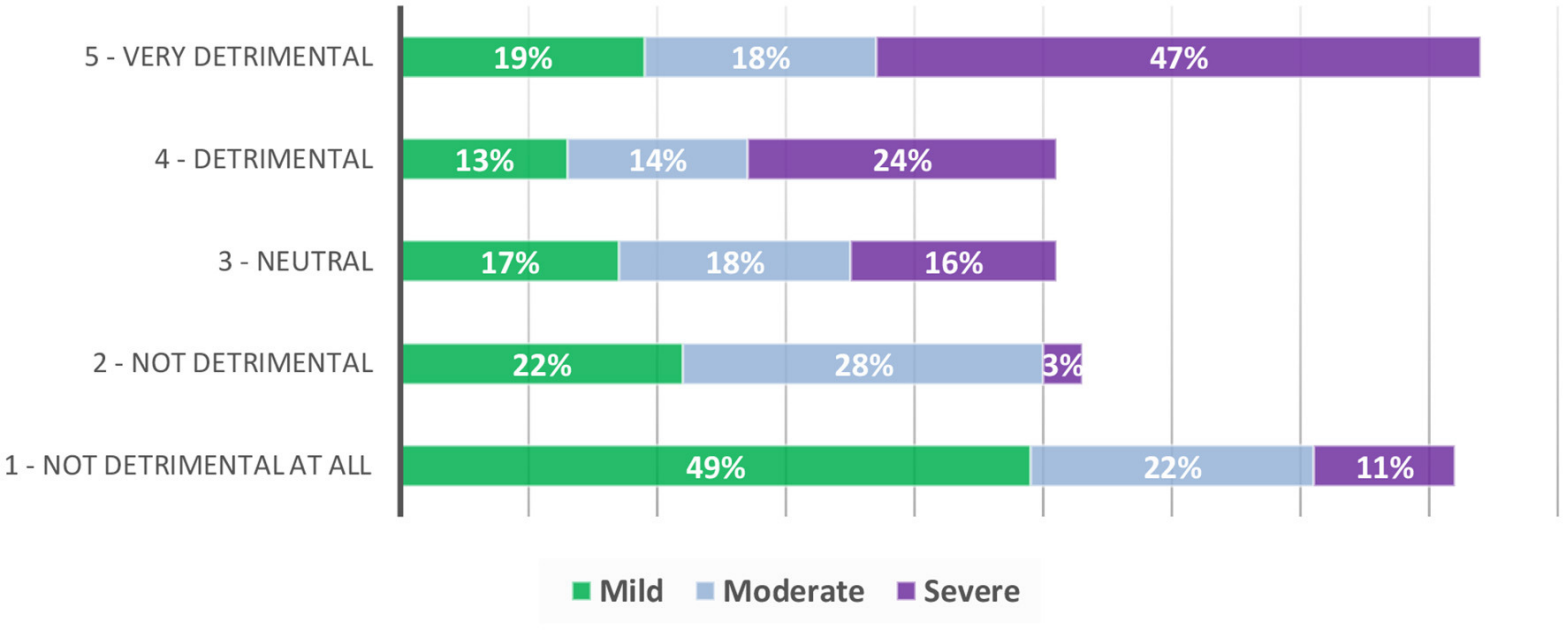
Perceived Impact of FAs on quality of life in data subjects with mild, moderate and severe FAs.

A greater proportion responded that FAs were detrimental to their overall quality of life, especially when they felt their FA treatment had been less or not at all successful [29% (CI: 18.6, 41.3) vs. 18% (CI: 11.5–26.2, see Table 3)]. Nearly half of data subjects who reported severe FAs (*n* = 38) reported that they had a detrimental impact on their quality of life (47%) (see Figure 2).

**Table 3:** Reported difficulty of having FAs and impact on everyday situations in each severity group.

	**How severe would you say your FAs are?**				
	**All patients and caregivers (*n* = 235)**	**Mild** **(*n* = 71)**	**Moderate (*n* = 120)**	**Severe (*n* = 38)**	**Unsure (*n* = 6)**
**Q9 On average, how troublesome is it having your FAs?**					
1 Not at all	25 (11%)	17 (24%)	6 (5%)	1 (3%)	1 (17%)
2	53 (23%)	23 (32%)	27 (22%)	3 (8%)	–
3	55 (23%)	21 (30%)	29 (24%)	2 (5%)	3 (50%)
4	57 (24%)	8 (11%)	37 (31%)	10 (26%)	2 (33%)
5 Extremely	45 (19%)	2 (3%)	21 (18%)	22 (58%)	–
**Q10.1 What impact have your FAs had on being at home?**					
1 Little to no impact	139 (59%)	52 (73%)	71 (59%)	15 (39%)	1 (17%)
2	42 (18%)	9 (13%)	28 (23%)	4 (11%)	1 (17%)
3	31 (13%)	5 (7%)	17 (14%)	7 (18%)	2 (33%)
4	12 (5%)	–	3 (2%)	8 (21%)	1 (17%)
5 Extremely high/negative impact	5 (2%)	1 (1%)	1 (1%)	3 (8%)	–
6 N/A	6 (3%)	4 (6%)	–	1(3%)	1 (17%)
**Q10.2 What impact have your FAs had on you being at school?**					
1 Little to no impact	50 (21%)	25 (35%)	22 (18%)	2 (5%)	1 (17%)
2	29 (12%)	10 (14%)	19 (16%)	–	–
3	28 (12%)	9 (13%)	14 (12%)	4 (11%)	1 (17%)
4	38 (16%)	7 (10%)	22 (18%)	7 (18%)	2 (33%)
5 Extremely high/negative impact	50 (21%)	8 (11%)	26 (22%)	15 (39%)	1 (17%)
6 N/A	40 (17%)	12 (17%)	17 (14%)	10 (26%)	1 (17%)
**Q10.3 What impact have your FAs had on college?**					
1 Little to no impact	45 (19%)	25 (35%)	19 (16%)	–	1 (17%)
2	17 (7%)	4 (6%)	11 (9%)	1 (3%)	1(17%)
3	15 (6%)	5 (7%)	6 (5%)	4 (11%)	–
4	22 (9%)	4 (6%)	13 (11%)	4 (11%)	1 (17%)
5 Extremely high/negative impact	35 (15%)	2 (3%)	20 (17%)	13 (34%)	–
6 N/A	101 (43%)	31 (44%)	51 (42%)	16 (42%)	3 (50%)
**Q10.4 What impact have your FAs had on university?**					
1 Little to no impact	49 (21%)	24 (34%)	22 (18%)	2 (5%)	1 (17%)
2	11 (5%)	5 (7%)	6 (5%)	–	–
3	10 (4%)	4 (6%)	3 (2%)	2 (5%)	1 (17%)
4	13 (6%)	1 (1%)	10 (8%)	–	2 (33%)
5 Extremely high/negative impact	14 (6%)	1 (1%)	10 (8%)	3 (8%)	–
6 N/A	138 (59%)	36 (51%)	69 (58%)	31 (82%)	2 (33%)
**Q10.5 What impact have your FAs had on work?**					
1 Little to no impact	47 (20%)	25 (35%)	19 (16%)	2 (5%)	1 (17%)
2	18 (8%)	6 (8%)	11 (9%)	–	1 (17%)
3	25 (11%)	10 (14%)	10 (8%)	4 (11%)	1 (17%)
4	24 (10%)	2 (3%)	16 (13%)	5 (13%)	1 (17%)
5 Extremely high/negative impact	26 (11%)	2 (3%)	18 (15%)	6 (16%)	–
6 N/A	95 (40%)	26 (37%)	46 (38%)	21 (55%)	2 (33%)
**Q10.6 What impact have your FAs had on social situations?**					
1 Little to no impact	43 (18%)	22 (31%)	20 (17%)	1 (3%)	–
2	32 (14%)	14 (20%)	17 (14%)	–	1 (17%)
3	39 (17%)	10 (14%)	21 (18%)	6 (16%)	2 (33%)
4	45 (19%)	12 (17%)	25 (21%)	7 (18%)	1 (17%)
5 Extremely high/negative impact	59 (25%)	7 (10%)	30 (25%)	21 (55%)	1 (17%)
6 N/A	17 (7%)	6 (8%)	7 (6%)	3 (8%)	1 (17%)

Impact of FAs in general and in specific contexts: at home, work, places of education and in social situations was assessed (see Table 4; Figure 2). Across the whole sample, 43% (CI: 36.6, 49.6) of data subjects rated their FAs as being at least somewhat troublesome (4 or 5 on the scale), associated with increased severity. Of the 16% of data subjects who reported severe FAs, 84% (CI: 68.5, 93.8) reported them as being very to extremely troublesome.

**Table 4:** Psycho-social impact of FAs and impact on quality of life in each severity group.

	**How severe would you say your FAs are?**				
	**All patients and caregivers (*n* = 235)**	**Mild** **(*n* = 71)**	**Moderate (*n* = 120)**	**Severe (*n* = 38)**	**Unsure (*n* = 6)**
**Q11.1 I feel embarrassed by my Fas**					
1 Strongly disagree	84 (36%)	33 (46%)	39 (32%)	9 (24%)	3 (50%)
2	28 (12%)	15 (21%)	11 (9%)	1 (3%)	1 (3%)
3	37 (16%)	10 (14%)	24 (20%)	2 (5%)	1 (17%)
4	29 (12%)	7 (10%)	17 (14%)	4 (11%)	1 (17%)
5 Strongly agree	57 (24%)	6 (8%)	29 (24%)	22 (58%)	–
**Q11.2 My FAs prevent me from making new friends**					
1 Strongly disagree	120 (51%)	52 (73%)	53 (44%)	13 (34%)	2 (33%)
2	42 (18%)	12 (17%)	26 (22%)	2 (5%)	2 (33%)
3	31 (13%)	3 (4%)	21 (18%)	5 (13%)	2 (33%)
4	22 (9%)	3 (4%)	9 (8%)	10 (26%)	–
5 Strongly agree	20 (9%)	1 (1%)	11 (9%)	8 (21%)	–
**Q11.3 My FAs attract unkind comments**					
1 Strongly disagree	76 (32%)	38 (54%)	33 (28%)	5 (13%)	–
2	42 (18%)	15 (21%)	22 (18%)	2 (5%)	3 (50%)
3	38 (16%)	6 (8%)	23 (19%)	6 (16%)	3 (50%)
4	36 (15%)	6 (8%)	20 (17%)	10 (26%)	–
5 Strongly agree	43 (18%)	6 (8%)	22 (18%)	15 (39%)	–
**Q11.4 My FAs attract unwanted attention**					
1 Strongly disagree	53 (23%)	30 (42%)	19 (16%)	4 (11%)	–
2	45 (19%)	15 (21%)	29 (24%)	–	1 (17%)
3	38 (16%)	12 (17%)	21 (18%)	2 (5%)	3 (50%)
4	47 (20%)	8 (11%)	31 (26%)	6 (16%)	2 (33%)
5 Strongly agree	52 (22%)	6 (8%)	20 (17%)	26 (68%)	–
**Q11.5 My FAs make me feel self-conscious**					
1 Strongly disagree	83 (35%)	40 (56%)	31 (26%)	9 (24%)	3 (50%)
2	26 (11%)	8 (11%)	16 (13%)	2 (5%)	–
3	34 (14%)	11 (15%)	19 (16%)	3 (8%)	1 (17%)
4	32 (14%)	5 (7%)	21 (18%)	4 (11%)	2 (33%)
5 Strongly agree	60 (26%)	7 (10%)	33 (28%)	20 (53%)	–
**Q11.6 My FAs stop me from finding new hobbies and/or doing my favorite hobbies**					
1 Strongly disagree	151 (64%)	58 (82%)	75 (62%)	14 (37%)	4 (67%)
2	33 (14%)	6 (8%)	20 (17%)	6 (16%)	1 (17%)
3	24 (10%)	4 (6%)	11 (9%)	8 (21%)	1 (17%)
4	16 (7%)	2 (3%)	5 (4%)	9 (24%)	–
5 Strongly agree	11 (5%)	1 (1%)	9 (8%)	1 (3%)	–
**Q11.7 My FAs influence what clothes I wear**					
1 Strongly disagree	148 (63%)	55 (77%)	76 (63%)	15 (39%)	2 (33%)
2	33 (14%)	8 (11%)	19 (16%)	4 (11%)	2 (33%)
3	22 (9%)	3 (4%)	13 (11%)	5 (13%)	1 (17%)
4	14 (6%)	3 (4%)	4 (3%)	6 (16%)	1 (17%)
5 Strongly agree	18 (8%)	2 (3%)	8 (7%)	8 (21%)	–
**Q11. 8 My FAs impact my holidays in a negative way**					
1 Strongly disagree	129 (55%)	50 (70%)	67 (56%)	11 (29%)	1 (17%)
2	28 (12%)	6 (8%)	19 (16%)	2 (5%)	1 (17%)
3	36 (15%)	6 (8%)	18 (15%)	9 (24%)	3 (50%)
4	26 (11%)	5 (7%)	10 (8%)	10 (26%)	1 (17%)
5 Strongly agree	16 (7%)	4 (6%)	6 (5%)	6 (16%)	–
**Q11.9 My FAs are uncomfortable/itchy**					
1 Strongly disagree	92 (39%)	43 (61%)	44 (37%)	4 (11%)	1 (17%)
2	32 (14%)	12 (17%)	19 (16%)	–	1 (17%)
3	43 (18%)	6 (8%)	25 (21%)	9 (24%)	3 (50%)
4	33 (14%)	6 (8%)	20 (17%)	6 (16%)	1 (17%)
5 Strongly agree	35 (15%)	4 (6%)	12 (10%)	19 (50%)	–
**Q11.10 My FAs are painful**					
1 Strongly disagree	121 (51%)	49 (69%)	65 (54%)	6 (16%)	1 (17%)
2	41 (17%)	12 (17%)	23 (19%)	5 (13%)	1 (17%)
3	39 (17%)	8 (11%)	17 (14%)	10 (26%)	4 (67%)
4	15 (6%)	1 (1%)	10 (8%)	4 (11%)	–
5 Strongly agree	19 (8%)	1 (1%)	5 (4%)	13 (34%)	–
**Q11.11 My FAs are detrimental to my overall quality of life**					
1 Strongly disagree	67 (29%)	35 (49%)	27 (22%)	4 (11%)	1 (17%)
2	52 (22%)	17 (24%)	33 (28%)	1 (3%)	1 (17%)
3	41 (17%)	11 (15%)	21 (18%)	6 (16%)	3 (50%)
4	30 (13%)	4 (6%)	17 (14%)	9 (24%)	–
5 Strongly agree	45 (19%)	4 (6%)	22 (18%)	18 (47%)	1 (17%)
**Q12 Overall, to what extent do your FAs impact on you participating in social activities (e.g. meeting friends for activities**,					
**hobbies, leisure or general socializing)?**					
No impact	110 (47%)	46 (65%)	54 (45%)	8 (21%)	2 (33%)
2	34 (14%)	13 (18%)	16 (13%)	2 (5%)	3 (50%)
3	36 (15%)	8 (11%)	23 (19%)	5 (13%)	–
4	35 (15%)	4 (6%)	16 (13%)	14 (37%)	1 (17%)
Extremely high impact	20 (9%)	–	11 (9%)	9 (24%)	–
**Q13 And how frequently would you typically want to participate in social activities (e.g. meeting friends for activities, hobbies**,					
**leisure or general socializing), but are unable to do so due to your FAs?**					
Always	20 (9%)	6 (8%)	10 (8%)	4 (11%)	–
Frequently	32 (14%)	3 (4%)	17 (14%)	12 (32%)	–
Sometimes	37 (16%)	5 (7%)	21 (18%)	10 (26%)	1 (17%)
Rarely	57 (24%)	18 (25%)	29 (24%)	5 (13%)	5 (83%)
Never	89 (38%)	39 (55%)	43 (36%)	7 (18%)	–

Over half of data subjects (59%, CI: 52.4, 65.4) reported that their FAs had little impact at home (see Table 3), however there was a trend toward increased impact in the presence of increased FA severity. While 73% (CI: 61.2, 82.9) of those with mild FAs reported no impact at home, only 39% (CI: 23.6, 56.2) with severe FAs reported no impact in this setting with nearly a fifth [21% (CI: 9.5, 37.3)] reporting an extremely high/very negative impact in this setting. The greatest negative impact was in response to social situations (see Table 3) with 44% of all subjects reporting a high or extremely high impact, this was followed by school (37% of data subjects reporting a high or extremely high impact); university (27% of data subjects reporting a high or extremely high impact); college (with 24% of data subjects reporting a high or extremely high impact) and work (with 21%) of data subjects reporting a high or extremely high impact. The effect of having FAs on social situations was extremely marked for those with severe FAs with 73% (CI: 56.2, 86.1) of data subjects with severe FAs reported a high or/extremely high negative impact on social situations (see Table 3).

The psychosocial impact of FAs and impact on quality of life was assessed (see Table 4). Similar to other categories, the psychosocial impacts of FAs appear to increase with increased severity. While the majority of data subjects with FAs (mild to severe) reported that their FAs had little to no impact on socializing (61%), making new friends (69%), finding/doing a favorite hobby (78%), style choices (77%) or impact on holidays (67%) this was not the case among the subgroup of those reporting severe FAs. In this group; 69% (CI: 52.0, 83.0) reported feeling embarrassed by them, as well as self-conscious (64%, CI: 46.8, 78.9) and reported receiving unkind comments (65%, CI: 47.9, 79.7) or unwanted attention (84%, CI: 68.5, 93.8).

Pain and discomfort from FAs were mostly reported in data subjects with severe FAs (see Table 4), with 66% (CI: 48.9, 80.5) reporting that their FAs feel uncomfortable/itchy and 45% (CI: 28.9, 62.0) reporting that their FAs were painful. The pattern of increased impact with increased FA severity also translated to the impact of FAs on overall quality of life, with 71% (CI: 54.0, 84.5) of those reporting severe FAs reporting that they are detrimental to their quality of life (Figure 2) compared to those with moderate (32%, CI: 23.8, 41.1) and mild FAs (12%, CI: 5.5, 21.9).

The impact of FAs on anxiety and depression was assessed (see Table 5). The frequency of data subjects reporting higher levels of anxiety (responding “rather much” or “very much” to Q14. “To what extent have you been feeling anxious during the last month?”) was highest in those with severe FAs (50%, CI: 33.4, 66.6), followed by those with moderate (31%, CI: 22.9, 40.1) and mild FAs (14%, CI: 6.9, 24.3). There was a similar association between increased feelings of depression and increased FA severity.

**Table 5:** Impact of FAs on anxiety and depression in each severity group.

	**How severe would you say your FAs are?**				
	**All patients and caregivers (*n* = 235)**	**Mild** **(*n* = 71)**	**Moderate (*n* = 120)**	**Severe (*n* = 38)**	**Unsure (*n* = 6)**
**Q14 To what extent have you been feeling anxious during the last month?**					
Not at all	42 (18%)	22 (31%)	17 (14%)	2 (5%)	1 (17%)
Only a little	59 (25%)	24 (34%)	25 (21%)	9 (24%)	1 (17%)
To some extent	64 (27%)	15 (21%)	40 (33%)	8 (21%)	1 (17%)
Rather much	42 (18%)	7 (10%)	22 (18%)	13 (34%)	-
Very much	28 (12%)	3 (4%)	16 (13%)	6 (16%)	3 (50%)
**Q15 To what extent have you been feeling sad or depressed during the last month?**					
Not at all	44 (19%)	26 (37%)	14 (12%)	3 (8%)	1 (20%)
Only a little	77 (33%)	27 (38%)	41 (34%)	8 (22%)	1 (20%)
To some extent	54 (23%)	9 (13%)	34 (29%)	10 (27%)	1 (20%)
Rather much	35 (15%)	6 (8%)	19 (16%)	9 (24%)	1 (20%)
Very much	22 (9%)	3 (4%)	11 (9%)	7 (19%)	1 (20%)

Caregivers completing the survey on behalf of the person with FAs were asked two further questions on how caring for that person impacted on their daily life (see Tables 6, 7). Most caregivers reported little impact on their own ability to work; find and participate in hobbies; go on holiday; or look after other family members (Q16). However, those caring for people with moderate and severe FAs reported higher levels of impact on socializing with 38% (CI: 28.5, 48.3) of the 100 caregivers of people with moderate or severe FAs having reported a high to extremely high impact on socializing.

**Table 6:** The impact of caring for someone with mild, moderate or severe FAs.

	**Severity of FAs in person cared for**				
	**All caregivers** **(*n* = 135)**	**Mild** **(*n* = 33)**	**Moderate** **(*n* = 70)**	**Severe** **(*n* = 30)**	**Unsure** **(*n* = 2)**
**Q16.1 Overall, to what extent do the facial angiofibromas of the person you care for impact you in: Meeting/socializing with my own friends**					
1 No impact	92 (68%)	24 (73%)	53 (76%)	13 (43%)	2 (100%)
2	16 (12%)	1 (3%)	11 (16%)	4 (13%)	–
3	9 (7%)	5 (15%)	1 (1%)	3 (10%)	–
4	9 (7%)	2 (6%)	3 (4%)	4 (13%)	–
5 Extremely high impact	9 (7%)	1 (3%)	2 (3%)	6 (20%)	–
**Q16.2 Overall, to what extent do the facial angiofibromas of the person you care for impact you in: Ability to work**					
1 No impact	104 (77%)	25 (76%)	59 (84)	18 (60%)	2 (100%)
2	11 (8%)	3 (9%)	4 (6%)	4 (13%)	–
3	9 (7%)	3 (9%)	5 (7%)	1 (3%)	–
4	4 (3%)	-	1 (1%)	3 (10%)	–
5 Extremely high impact	7 (5%)	2 (6%)	1 (1%)	4 (13%)	–
**Q16.3 Overall, to what extent do the facial angiofibromas of the person you care for impact you in: Participate in my own hobbies/find new hobbies**					
1 No impact	102 (76%)	25 (76%)	57 (81%)	18 (60%)	2 (100%)
2	9 (7%)	3 (9%)	4 (6%)	2 (7%)	–
3	4 (3%)	1 (3%)	2 (3%)	1 (3%)	–
4	12 (9%)	1 (3%)	6 (9%)	5 (17%)	–
5 Extremely high impact	8 (6%)	3 (9%)	1 (1%)	4 (13%)	–
**Q16.4 Overall, to what extent do the facial angiofibromas of the person you care for impact you in: Going on holiday**					
1 No impact	89 (66%)	24 (73%)	49 (70%)	14 (47%)	2 (100%)
2	10 (7%)	–	9 (13%)	1 (3%)	–
3	10 (7%)	3 (9%)	4 (6%)	3 (10%)	–
4	15 (11%)	4 (12%)	6 (9%)	5 (17%)	–
5 Extremely high impact	11 (8%)	2 (6%)	2 (3%)	7 (23%)	–
**Q16.5 Overall, to what extent do the facial angiofibromas of the person you care for impact you in: Look after other family members**					
1 No impact	100 (74%)	25 (76%)	58 (83%)	15 (50%)	2 (100%)
2	10 (7%)	4 (12%)	4 (6%)	2 (7%)	–
3	12 (9%)	2 (6%)	5 (7%)	5 (17%)	–
4	8 (6%)	–	3 (4%)	5 (17%)	–
5 Extremely high impact	5 (4%)	2 (6%)	–	3 (10%	–
**Q17.1 How frequently would you want to participate in but were unable to do so: Meeting/socializing with my own friends**.					
Never	77 (58%)	19 (58%)	44 (64%)	13 (45%)	1 (100%)
Rarely	11 (8%)	3 (9%)	5 (7%)	3 (10%)	–
Sometimes	18 (14%)	2 (6%)	9 (13%)	7 (24%)	–
Frequently	17 (13%)	5 (15%)	6 (9%)	6 (21%)	–
Always	9 (7%)	4 (12%)	5 (7%)	–	–
**Q17.2 How frequently would you want to participate in but were unable to do so: Ability to work**.					
Never	85 (64%)	21 (64%)	47 (68%)	16 (55%)	1 (100%)
Rarely	11 (8%)	1 (3%)	6 (9%)	4 (14%)	–
Sometimes	11 (8%)	1 (3%)	5 (7%)	5 (17%)	–
Frequently	12 (9%)	4 (12%)	4 (6%)	4 (14%)	–
Always	13 (10%)	6 (18%)	7 (10%)	–	–
**Q17.3 How frequently would you want to participate in but were unable to do so: Participate in my own hobbies/find new hobbies**.					
Never	78 (59%)	19 (58%)	45 (65%)	13 (45%)	1 (100%)
Rarely	12 (9%)	2 (6%)	7 (10%)	3 (10%)	–
Sometimes	15 (11%)	2 (6%)	5 (7%)	8 (28%)	–
Frequently	16 (12%)	4 (12%)	7 (10%)	5 (17%)	–
Always	11 (8%)	6 (18%)	5 (7%)	–	–
**Q17.4 How frequently would you want to participate in but were unable to do so: Going on holiday**.					
Never	81 (61%)	22 (67%)	47 (68%)	11 (38%)	1 (100%)
Rarely	10 (8%)	1 (3%)	3 (4%)	6 (21%)	-
Sometimes	13 (10%)	–	7 (10%)	6 (21%)	–
Frequently	15 (11%)	4 (12%)	6 (9%)	5 (17%)	–
Always	13 (10%)	6 (18%)	6 (9%)	1 (3%)	–
**Q17.5 How frequently would you want to participate in but were unable to do so: Look after other family members**.					
Never	82 (62%)	20 (61%)	46 (67%)	15 (52%)	1 (100%)
Rarely	9 (7%)	2 (6%)	5 (7%)	2 (7%)	–
Sometimes	16 (12%)	3 (9%)	8 (12%)	5 (17%)	–
Frequently	14 (11%)	3 (9%)	5 (7%)	6 (21%)	–
Always	11 (8%)	5 (15%)	5 (7%)	1 (3%)	–

**Table 7:** Percentage of caregivers reporting a high level of impact across the situations assessed in questions 16 and 17.

**Q16: Impact of FAs in various situations** **(1 = no impact−5 = extremely high impact)**	**% scoring** **4 or 5**
Meeting/socializing with my own friends	13%
Ability to work	8%
Participate in my own hobbies/find new hobbies	15%
Going on holiday	19%
Look after other family members	10%
**Q17: Frequency of being unable to participate in various activities (from** **never to always)**	**% scoring frequently/always**
Meeting/socializing with my own friends	20%
Ability to work	19%
Participate in my own hobbies/find new hobbies	20%
Going on holiday	21%
Look after other family members	19%

Some caregivers, particularly those who cared for people with severe FAs, reported not being able to do certain activities as frequently as they would like (see Tables 6, 7, Q17). Forty-five percent (CI: 26.9, 64.1) of those who cared for people with severe FAs reported not being able to participate in social activities as often as they would like for at least some of the time (having selected either “sometimes,” “frequently,” or “always”). Most responses highlighted the broad impact of FAs for carers in various situations, showing that in the region of a fifth reported experiencing a “high” or “extremely” negative impact in a range of situations and activities such as socializing, working, participating in hobbies, going on holiday and looking after other family members.

## Discussion

This was a cross-sectional study conducted in the UK and Germany with the aim of exploring the impact of living with FAs for those affected by TSC as well as for their caregivers and wider family. This survey supports previous evidence that FAs first appear at a young age of 3–5 years (6), younger than has been previously reported (5).

More participants with moderate and severe FAs reported a negative impact in their day-to-day lives, at work, places of education and in social situations in general, compared to those with mild FAs. Most participants with severe FAs reported negative impacts such as feeling embarrassed (69%), self-conscious (64%), receiving unkind comments (65%) or attracting unwanted attention (84%). In terms of physical impacts, 66% of those with severe FAs reported that they were uncomfortable/itchy and of these, nearly half reported they were painful. Seventy-one percent of those with severe FAs reported that they are detrimental to their quality of life (see Figure 2) compared to those with moderate (32%) and mild FAs (12%). Additionally, those who cared for TSC affected individuals with moderate and severe FAs reported greater negative impacts than those who cared for those with mild FAs.

Aside from those affected by severe FAs, the impact on quality of life was broadly reported by 36% of data subjects in the UK and 28% of data subjects in Germany, reflecting a common negative impact from this widely prevalent feature of TSC. The current findings demonstrate a substantial burden of TSC on the personal lives of individuals with TSC and their families (14) and the negative psychosocial and mental health impacts of living with FAs. While FAs are a common feature of TSC, this survey demonstrates that for the 16% of those who described having severe FAs, the negative impact cuts across a broad spectrum of daily life, in particular psychosocial categories. The high prevalence of reported negative experiences among affected by severe FAs (38/235 data subjects) is cause for concern.

**Figure 3:**
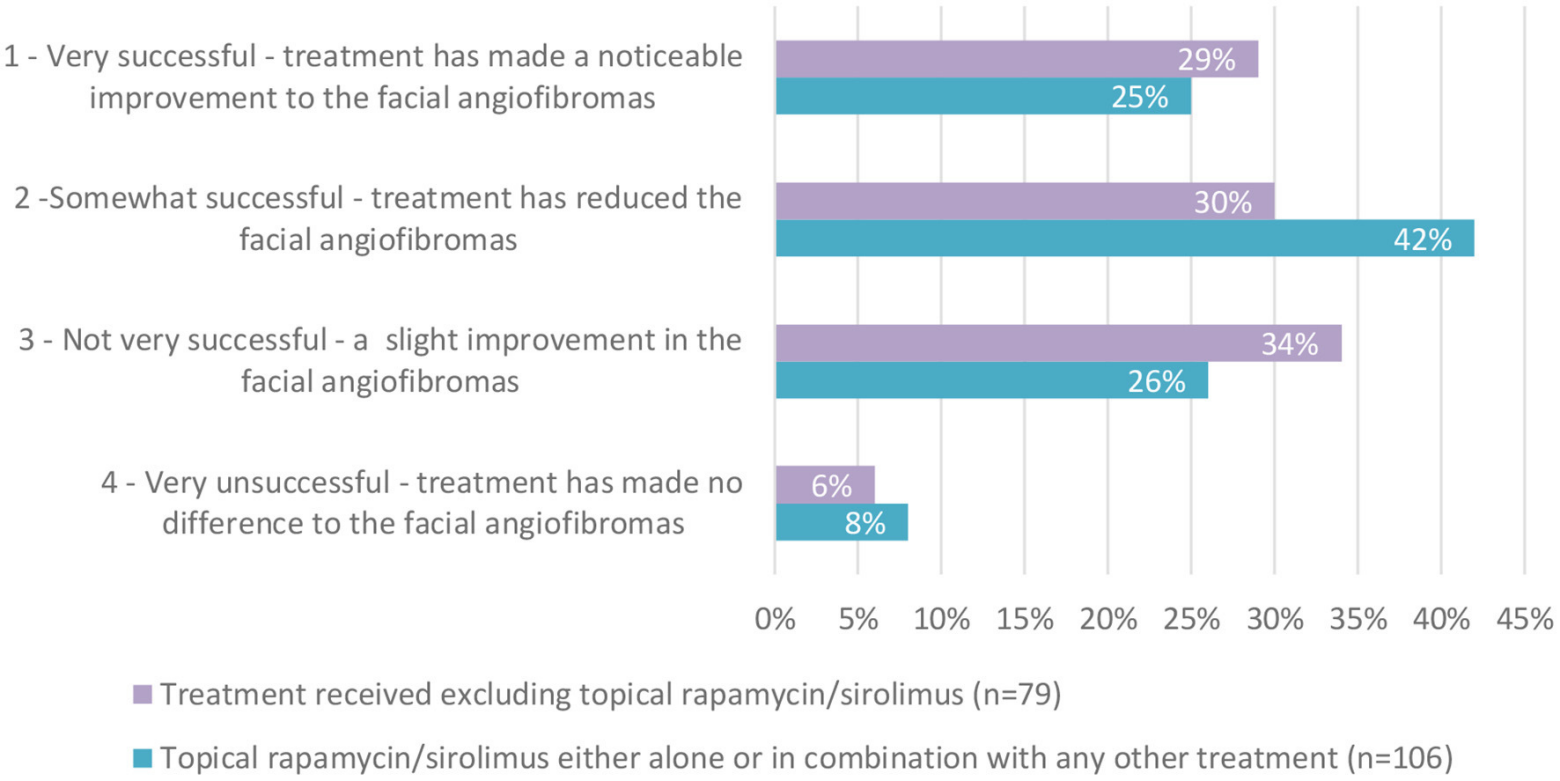
Perceived efficacy of treatment regimens for FAs.

The survey explored current treatment options and identified that the most common treatment, topical rapamycin/sirolimus, either alone or in combination, was reported to be of benefit to most participants, successfully treating and helping their FAs either somewhat or very successfully in 67% (Figure 3).

## Limitations

The survey received a limited number of completed responses with a combined total of 762 individuals accessing the survey, 24 failing to meet screening criteria and 503 individuals not completing the survey to at least question 14. In view of the large number of individuals believed to have a diagnosis of TSC, estimated to be between 3,700 and 11,000 in the UK alone, according to the UK Tuberous Sclerosis Association, further studies with a larger response rate from individuals with TSC or their caregivers would be needed to confirm this study's preliminary findings.

As with all self-completed surveys, only data from those people willing to participate were captured, therefore a bias of self-selected population reporting is expected. Among the data subjects, 40% were patients (94 patients and 141 caregivers contributed data, 235 in total) therefore a greater proportion were represented by proxy.

Data was self-reported (e.g., in responses to screening questions, grading of symptoms and impacts) and subjective with no validation of responses against clinical records. The survey did not assess the medical background of the TSC affected individuals, in particular the prevalence of TSC-associated Neuropsychiatric Disorders (TAND) of which includes anxiety and depressive disorders. The prevalence of these has previously been reported as: anxiety disorders (28%) and mood disorders (26%) in a survey of 241 children and adults with TSC (17). It is possible that TAND could influence responses relating to psychosocial aspects of the survey and equally, that the presence of mild to severe FAs may have a varying degree of impact for those affect by TAND.

## Conclusion

FAs are a visible manifestation of TSC and may mimic severe forms of acne vulgaris, a condition which has well-described negative impacts on stress, interpersonal relations and daily life (18). FAs are present on the most socially engaged aspect of the body; the face, and incur negative psychosocial impacts (19). They may be considered an example of a psychodermatological condition. In this survey, we found the impact of FAs to be comparable to previous findings on the psychosocial impacts reported by those with acne vulgaris, namely that subjective ratings of severity of acne vulgaris are related to reported negative self-image, self-esteem, social relations and depression scores (18). Studies focussed on the psychosocial impacts of acne vulgaris suggest that the reported impacts are independent of objective measures, encouraging clinicians to focus on the subjective perception in managing the condition, irrespective of objective measures of severity (18).

The results from this survey quantify the perceived social and physical impacts of FAs as well as the perceived efficacy of their treatment options, as reported by affected patients or caregivers by proxy, in the UK and Germany. This is the first survey assessing the impact of FAs not only on the patient group but also on the working and social lives of their caregivers. The findings are notable for the high proportion of data subjects reporting a negative impact of FAs on affected individuals' day-to-day activities, leading to large numbers reporting a detrimental impact on their overall quality of life, particularly among those reporting severe FAs. For the caregivers, caring for someone with with FAs did not appear to hinder the ability of most participating carers to socialize, work, find and participate in hobbies, go on holiday and look after other family members. However, similar to the answers provided in the main survey, generally the frequency of no/low impact was higher in caregivers if the person they cared for had mild FAs, whereas those who cared for people with moderate and severe FAs reported more of an impact.

This survey highlights the wide-ranging and often severe impact of FAs on both physical and social experience and the need to ensure adequate efforts are made to provide effective treatment. Clinicians should consider proactive assessment and treatment of facial angiofibromas with available treatments, which may include topical rapamycin/sirolimus among other therapeutic options, such as laser ablation and electro-dissection.

## Data availability statement

The original contributions presented in the study are included in the article/supplementary material, further inquiries can be directed to the corresponding author.

## Ethics statement

The studies involving human participants were reviewed and approved by Western Institutional Review Board (Tracking number: 20214481). Written informed consent to participate in this study was provided by the participants' legal guardian/next of kin.

## Author contributions

MM authored the manuscript and implemented editorial changes following the feedback. SA critically revised the manuscript and approved the final version to be published. MK contributed to the conception/design of the study, contributed to the acquisition, analysis or interpretation of data, critically revised the manuscript, and approved the final version to be published. PT and LL contributed to the design, dissemination of the study, critically reviewed, revised the manuscript, and approved the final version to be published. All authors contributed to the article and approved the submitted version.

## Conflict of interest

This study was commissioned by Plusultra pharma, David Jones and Mark Partington. The report was produced by Adelphi Real World. The Tuberous Sclerosis Association (TSA) has received sponsorship for events from PlusUltra Pharmaceuticals. MM is completing a study with a salary funded by PTC Therapeutics. No personal financial relation to pharmaceutical companies. SA has received funding from GW Pharmaceuticals, Norvartis, PTC Therapeutics, Boston Scientific, Nutricia, UCB, BioMarin, LivaNova, Medtronic, Desitin, Ipsen, CDKL5 UK, TSA and the National Institute for Health Research. The remaining authors declare that the research was conducted in the absence of any commercial or financial relationships that could be construed as a potential conflict of interest.

## Publisher's note

All claims expressed in this article are solely those of the authors and do not necessarily represent those of their affiliated organizations, or those of the publisher, the editors and the reviewers. Any product that may be evaluated in this article, or claim that may be made by its manufacturer, is not guaranteed or endorsed by the publisher.

## Abbreviations

EphMRA: European Pharmaceutical Market Research Association
FA: Facial Angiofibroma
GDPR: General Data Protection Regulations
mTOR: mammalian (or “Mechanistic”) Target of Rapamycin
PO: Patient Organisation
TAND: TSC Associated Neuropsychiatric Disorders
TSC: Tuberous Sclerosis Somplex
UK: United Kingdom
WIRB: Western Institutional Review Board.
